# Dromedary camel CD14^high^ MHCII^high^ monocytes display inflammatory properties and are reduced in newborn camel calves

**DOI:** 10.1186/s12917-020-02285-8

**Published:** 2020-02-18

**Authors:** Jamal Hussen, Turke Shawaf, Abdullah I. A. Al-Mubarak, Naser Abdallah Al Humam, Faisal Almathen, Hans-Joachim Schuberth

**Affiliations:** 1 Department of Microbiology and Parasitology, College of Veterinary Medicine, King Faisal University, Al-Ahsa, 31982 Saudi Arabia; 2 Department of Clinical Studies, College of Veterinary Medicine, King Faisal University, Al-Ahsa, Saudi Arabia; 3 Department of Veterinary Public Health and Animal Husbandry, College of Veterinary Medicine, King Faisal University, Al-Ahsa, Saudi Arabia; 4 The Camel Research Center, King Faisal University, Al-Ahsa, Saudi Arabia; 5 Immunology Unit, University of Veterinary Medicine Hannover, Foundation, Hannover, Germany

**Keywords:** Monocyte subsets, Dromedary camel, Flow cytometry, Phagocytosis, Adhesion molecules

## Abstract

**Background:**

In human and different animal species, blood monocytes are classified based on their expression pattern of different monocytic markers into phenotypically and functionally different subsets. In the current study, we used flow cytometry and monoclonal antibodies to CD172a, CD14, CD163 and MHCII to identify monocyte subsets in peripheral blood of dromedary camels.

**Results:**

Based on CD14, CD163 and MHCII expression, camel CD172a + monocytes were divided into three subsets: The major subpopulation of camel monocytes (mo-I) showed high expression of CD14 and CD163, but low expression of MHCII. A second subset of monocytes (mo-II) expressed highly all three markers, CD14, CD163 and MHCII. A third monocyte subset (mo-III) displayed low expression of CD14 and CD163 with high MHCII expression. While the two MHCII^high^ subsets (mo-II and mo-III) showed higher expression of CD11a in comparison to the MHCII^low^ subset (mo-I), CD18 and CD11b were highest expressed on the two CD14^high^ subsets (mo-I and mo-II). Bacterial stimulation of camel leukocytes identified mo-II cells as an antimicrobial monocyte subset with the highest phagocytic and ROS production capacity. The comparison of monocyte counts and phenotype between newborn calves and adult camels revealed significantly reduced numbers of mo-II cells in newborn animals. Monocytes of newborns expressed significantly more CD172a and CD163 molecules but less CD14 and MHCII molecules than monocytes of adult camels.

**Conclusions:**

Camel monocyte subsets, mo-I, mo-II and mo-III are counterparts of bovine classical, intermediate and non-classical monocytes respectively. The distribution of camel monocyte subsets is influenced by age.

## Background

Blood monocytes are innate immune cells with essential role in the defense against pathogens [1]. Monocytes are equipped with a vast array of receptors that mediate pathogen recognition, phagocytosis and subsequent production of reactive oxygen species (ROS) [2, 3].

Studies in humans [4], mice [5], cows [6–8], pigs [9] and dogs [10] have classified monocytes into different subsets with subset-specific phenotype and function [11]. In human and cattle, monocytes were classified based on their CD14 and CD16 expression into CD14^++^ CD16^−^ classical monocytes (90% of total monocytes), CD14^++^ CD16^+^intermediate monocytes (5% of total monocytes) and CD14^+^ CD16^++^ non-classical monocytes (5% of total monocytes) [7, 12]. However, in species, where CD14 expression is low, like in the mouse, [5], or where no cross-reactive antibodies against CD16 are available, like in the dog [10], other surface molecules have been used for the classification of blood monocytes. In the mouse, the myeloid markers Ly6C and CD43 were used for the analysis of monocyte heterogeneity [13]. The identification of monocyte subsets in the pig was based on the differential expression of CD14 and CD163 [9, 14]. Dog monocytes were divided into three monocyte subsets based on CD14 and MHCII molecules expression patterns [10].

Monocyte subsets showed phenotypic and functional differences in a species-specific manner. This includes the expression of different monocytic markers, cell adhesion molecules, cytokines and chemokine receptors [11, 15]. In addition, many functional differences have been identified between monocyte subsets. Human and bovine CD14^high^ monocytes, including classical and intermediate monocytes, showed enhanced anti-bacterial activity, including phagocytosis and ROS generation capacity [6, 12], whereas a patrolling function along the endothelium and a role in anti-viral immunity have been described for human and mouse CD14^low^ monocytes (non-classical monocytes) [16].

To our knowledge, there are no studies on the heterogeneity of blood monocytes in dromedary camels. Therefore, this study aimed at the analysis of phenotype and function of camel blood monocytes and the identification of monocyte subsets in newborn and adult camels.

## Results

### Phenotypic characterization of camel monocytes

**Fig. 1:**
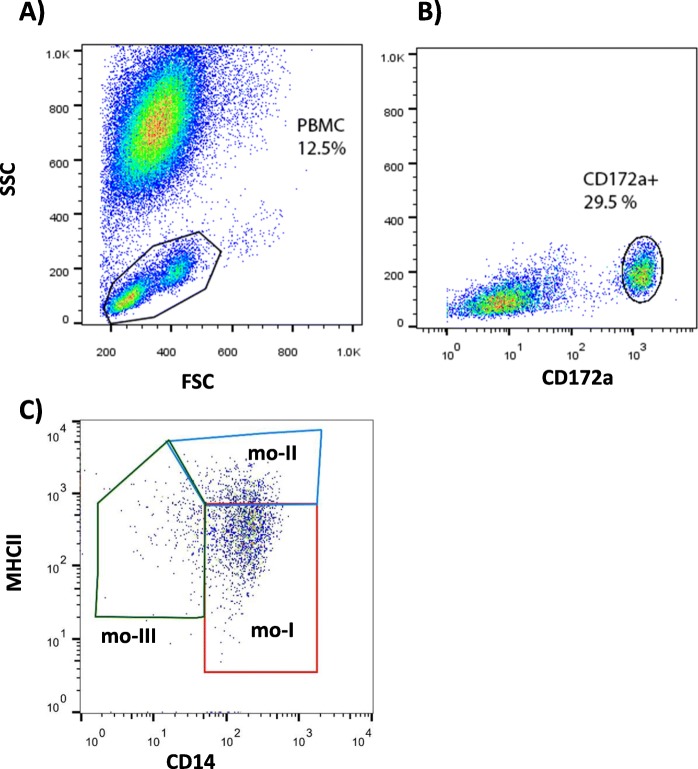
Gating strategy of camel monocyte subsets. Camel leukocytes were separated by hypotonic lysis of erythrocytes and separated cells were labeled with monoclonal antibodies to CD14, CD172a and MHCII molecules and analyzed by flow cytometry. A) Mononuclear cells (PBMC, encircled region) were identified based on their forward (FSC) and side scatter (SSC) properties. B) In a SSC vs CD172a dot plot of gated PBMC, a region was set on CD172a-positive monocytes). C) Correlated dot plots of CD14 versus MHCII fluorescence after gating on CD172a-positive cells. Monocyte subsets mo I, II, and III were identified according to their CD14 and MHCII expression density (mo-I: CD14^high^MHCII^low^, mo-II: CD14^high^MHCII^high^, mo-III: CD14^low^MHCII^high^)

**Fig. 2:**
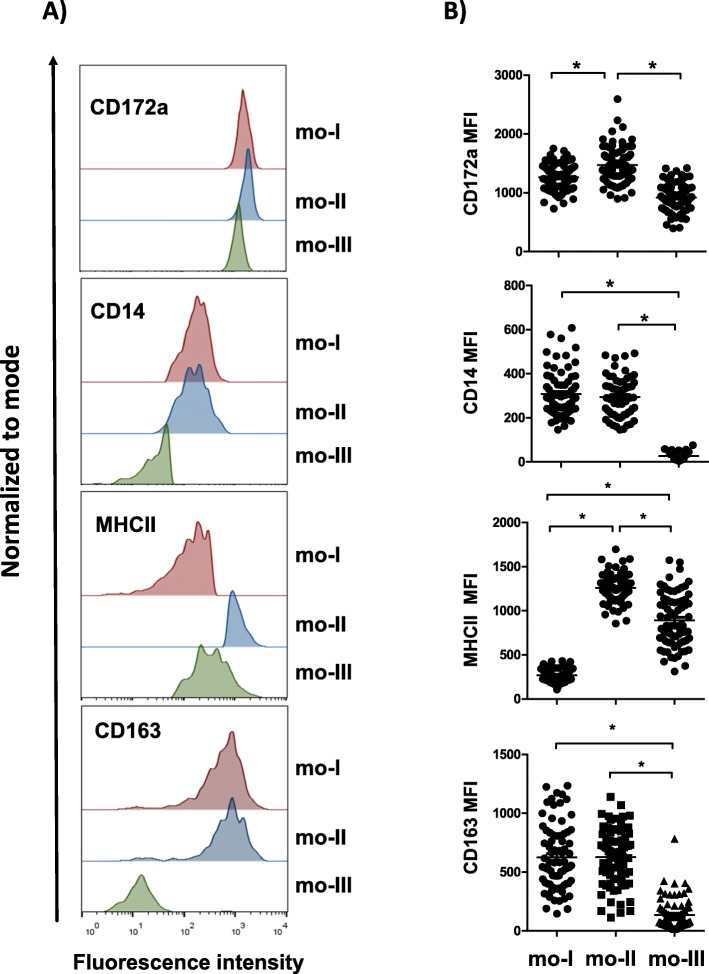
Differential expression of cell surface molecules on camel monocyte subsets. (A) Separated camel leukocytes were labelled with monoclonal antibodies to CD14, CD172a, MHCII and CD163 in different combinations and were analysed by flow cytometry. After setting gates on camel monocyte subsets (based on CD14 and MHCII expression), expression levels of CD14, CD172a, MHCII and CD163 are shown as histograms. (B) Comparison of mean fluorescence intensities between the camel monocyte subsets mo-I, mo-II and mo-III of analysed markers were graphically displayed. * indicates a significant difference (*p* value < 0.05) between groups as analysed by the one-way analysis of variance (ANOVA)

Camel monocytes were identified based on their FSC/SSC characteristics (Fig. 1a) and CD172a expression (Fig. 1b). Since no tested antibody specific for CD16 cross-reacted with camel CD16 (data not shown), antibodies specific for CD14, CD163 and MHCII were used to identify camel monocyte subsets. Based on the cell surface expression of CD14 and MHCII, three monocyte subsets were defined after flow cytometry (Fig. 1c). The major subpopulation of camel monocytes (mo-I) showed high expression of CD14 and CD163, but low expression of MHCII (CD14^high^CD163^high^MHCII^low^) (Fig. 2). A second subset of monocytes (mo-II) expressed all three markers at a high density (CD14^high^CD163^high^MHCII^high^). A third monocyte subset (mo-III) displayed low expression of CD14 and CD163 with high MHCII expression (CD14^low^CD163^low^MHCII^high^) (Fig. 2), which was higher than MHCII expression on mo-I but lower than MHCII expression on mo-II (Fig. 2b). Although all subsets expressed high levels of CD172a, the expression level of CD172a was significantly higher on mo-II cells compared with mo-I and mo-II cells (Fig.2b).

### Size and complexity of camel monocyte subsets

**Fig. 3:**
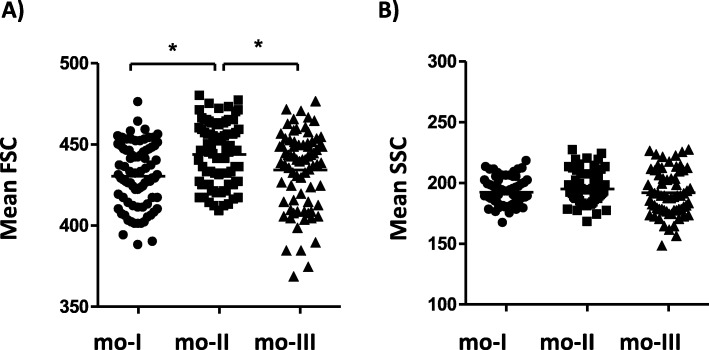
Size and complexity of camel monocyte subsets. Camel leukocytes were labelled with monoclonal antibodies to CD14, CD172a and MHCII and were analysed by flow cytometry. After gating on camel monocyte subsets mo-I, mo-II, and mo-III, their mean size (A, forward scatter, FSC) and mean complexity (B, side scatter, SSC) was determined and graphically displayed. * indicates a significant difference (p value < 0.05) between groups as analysed by the one-way analysis of variance (ANOVA)

The flow cytometrically determined mean forward scatter (correlated with size) and the mean side scatter (correlated with complexity) of camel monocyte subsets revealed, that camel mo-II cells were significantly larger in size when compared to mo-I and mo-II cells (Fig. 3a). The three monocyte subsets displayed no significant differences in their complexity (Fig. 3b).

### Distribution of monocyte subsets in blood of dromedary camel

With 86.4% ± 0.7% among CD172a + monocytes, camel mo-I represented the main monocyte population in blood, whereas the other two subsets mo-II (6.8% ± 0.3%) and III (5.0% ± 0.3%) represented minor fractions among blood monocytes (Table 1).

**Table 1:** Relative and absolute distribution of monocyte subsets in camel peripheral blood

	CD14^high^MHCII^low^	CD14^high^MHCII^high^	CD14^low^MHCII^high^
% of all monocytes	86.4 ± 0.7	6.8 ± 0.3	5.0 ± 0.3
Cells/ml blood (× 10^3^)	627.8 ± 27.1	80.1 ± 7.1	48.4 ± 5.0

Means ± SEM, Blood of 60 animals was analyzed

### Camel monocytes differ in their expression pattern of cell adhesion molecules

**Fig. 4:**
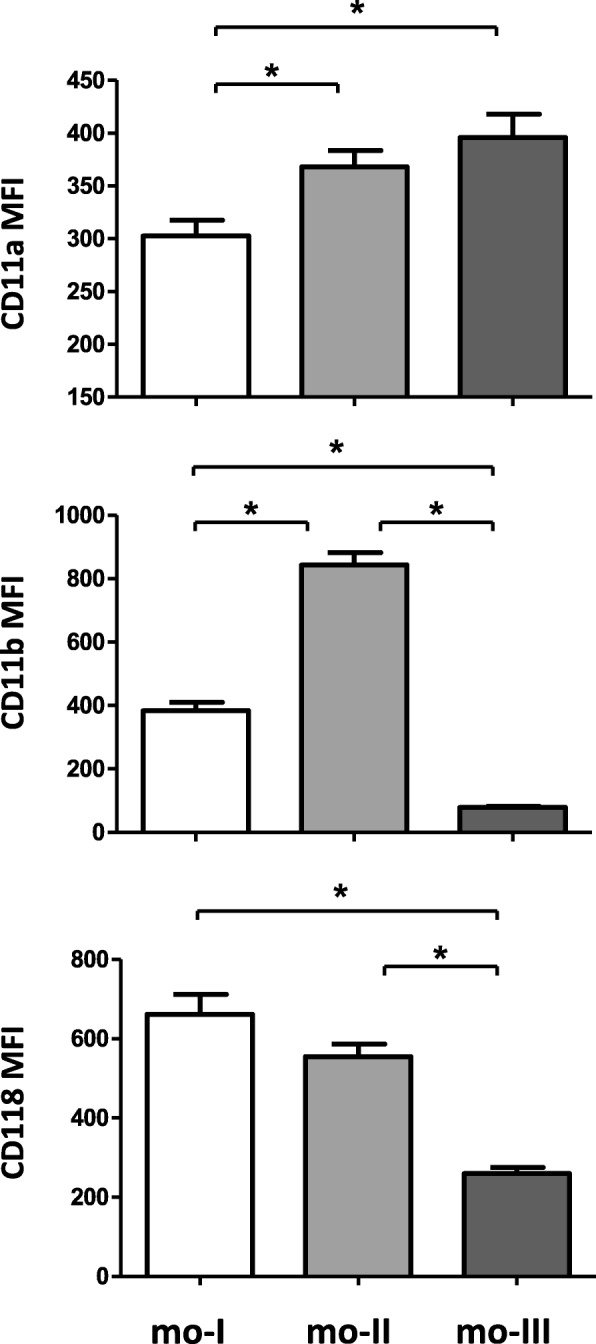
Expression densities of cell surface adhesion molecules on camel monocyte subsets. Separated camel leukocytes were labelled with monoclonal antibodies to CD14, MHCII, CD11a, CD11b or CD18 and were analysed by flow cytometry. After gating on camel monocyte subsets (mo-I, mo-II, mo-III), expression levels of CD11a, CD11b and CD18 were calculated as mean fluorescence intensities (mean ± SEM, *n* = 62 animals). * indicates a significant difference (p value < 0.05) between groups as analysed by the one-way analysis of variance (ANOVA)

While the two MHCII^high^ subsets (mo-II and mo-III) showed higher expression of CD11a in comparison to the MHCII^low^ subset (mo-I), CD18 was highest expressed on the two CD14^high^ subsets. CD14^high^/MHCII^high^ monocytes showed the highest expression of CD11b. However CD11b expression was higher on mo-I compared to the mo-III subset (Fig. 4).

### Camel monocyte subsets differ in their antimicrobial activity

**Fig. 5:**
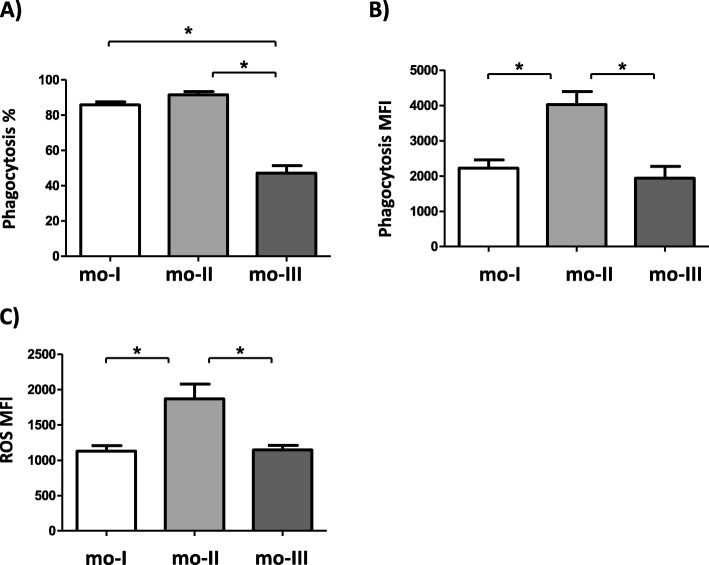
Phagocytosis and ROS-production capacity of camel monocyte subsets. Separated camel leukocytes were labeled with monoclonal antibodies to CD14 and MHCII. Labeled cells were incubated with heat killed FITC-labeled *S. aureus* bacteria. After gating on camel monocyte subsets (mo-I, mo-II, mo-III) the percentage of FITC-positive, phagocytosis-positive cells (A) and the mean fluorescence intensities (MFI) of phagocytosis-positive cells (B) was determined for each subset (means ± SEM). (C) Parallel setups of labeled camel leukocytes loaded with the ROS-sensitive dye dihydrorohdamin-123 (DHR-123) were stimulated with heat killed *S. aureus* bacteria. The reactive oxygen-dependent generation of rhodamin 123 was recorded flow cytometrically for gated camel monocyte subsets as the mean cellular fluorescence (ROS MFI, mean ± SEM, *n* = 21 animals.). Differences between groups were calculated using the one-way ANOVA and were considered significant (*) if *p* < 0.05

Antimicrobial activity of camel monocyte subsets was investigated by the capacity of each subset to ingest bacteria (phagocytosis) and to produce reactive oxygen species upon bacterial stimulation in vitro. Among the two CD14^high^ subsets (mo-I, mo-II), the percentage of phagocytosis-positive cells was about twice higher than for the CD14^low^ subset (mo-III) (Fig. 5a). The mean fluorescence intensity of phagocytosis-positive cells as an indicator for the number of bacteria phagocytosed per cell was highest for the CD14^high^MHCII^high^ subset (mo-II) (Fig. 5b). ROS production activity after bacterial stimulation followed the same pattern with mo-II cells showing significantly more ROS production compared to mo-I and mo-III monocytes (Fig. 5c).

### Monocyte subsets in newborn and adult camels differ in composition and phenotype

**Fig. 6:**
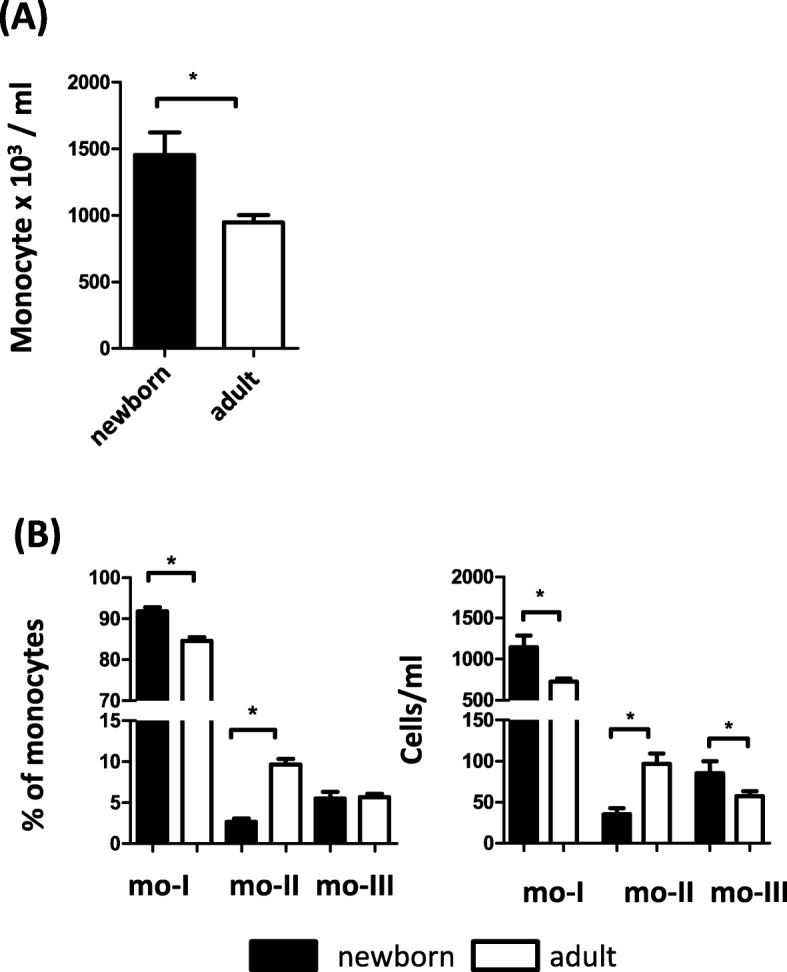
Monocyte subsets in newborn and adult camels. (A) Total numbers of monocytes/ ml blood after microscopic counting of total leukocytes and flow cytometric determination of monocyte percentages among leukocytes (mean ± SEM, n = 62 animals). Difference between groups was calculated using the t-test and was considered significant (*) if *p* < 0.05. (B) Percentages and cell counts / ml blood of three camel monocyte subsets (mo-I, mo-II, mo-III) of newborn (*n* = 23 animals) and adult (n = 62 animals) camels were calculated and presented as mean ± SEM. Difference between groups was calculated using the t-test and was considered significant (*) if *p* < 0.05

The total number of circulating monocytes in blood was significantly higher in newborn (< one month of age) camel calves (1453 ± 169 × 10^3^ cell/ml) when compared with monocyte number in adult (4–10 years) camels (947 ± 53 × 10^3^ cell/ml) (Fig. 6a). Among all monocytes, the fraction as well as the absolute numbers of mo- I monocytes were significantly higher in newborns than in adults (Fig. 6b). Although the fraction of mo-III cells among all monocytes was comparable between newborns and adults, absolute mo-III numbers were significantly higher in newborns (Fig. 6b). Compared with adult camels, newborns showed up with significantly lower numbers of mo-II and a significantly lower percentage of mo-II cells among all monocytes (Fig. 6b).

**Fig. 7:**
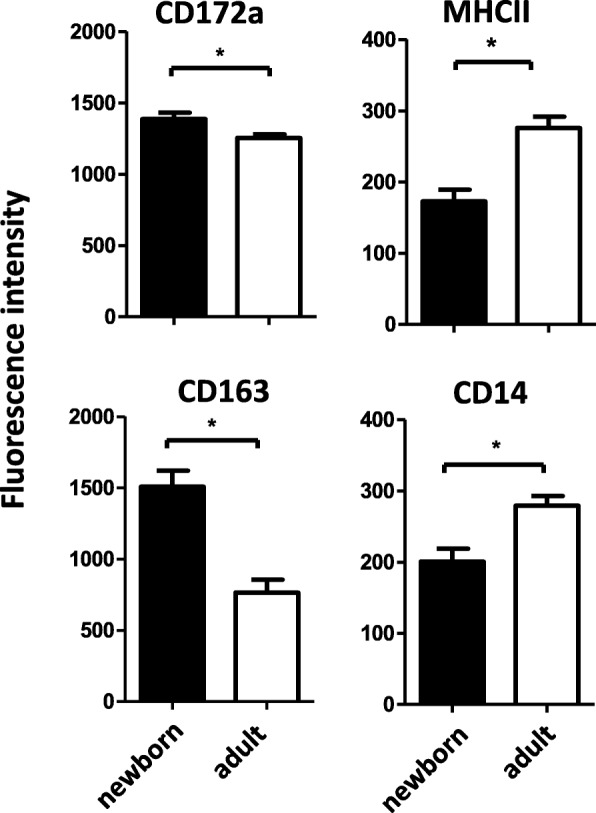
Expression densities of cell surface molecules on newborn and adult camel monocytes. Separated camel leukocytes were labeled with monoclonal antibodies to CD14, CD172a, MHCII and CD163 and analyzed by flow cytometry. After gating on camel monocyte subsets, expression levels of CD14, CD172a, MHCII and CD163 were measured as mean fluorescence intensities of analyzed markers and were shown as mean ± SEM. * indicates a significant difference (*p* value < 0.05) between groups as analyzed by the t-test

Comparing the density of expressed monocyte surface molecules, monocytes of newborn camels significantly expressed more CD172a and CD163 molecules whereas the expression of CD14 and MHCII was significantly lower than on monocytes of adult camels (Fig. 7).

## Discussion

Studies in different species revealed that monocytes are a heterogeneous population of innate immune cells consisting of phenotypically and functionally different subsets [4–11]. The clinical relevance of distinct monocyte subsets has been recently demonstrated for different species by linking the distribution of blood monocyte subsets to the susceptibility to different infectious [17] and non-infectious diseases [18, 19].

Although in most studied species monocyte subsets were identified based on the expression of the surface markers CD14 and CD16 [7, 12], other surface markers were used for monocyte classification in some species due to low CD14 expression [5] or unavailability of CD16 antibodies [10]. As no cross-reactive antibodies to camel CD16 were available [20], we used monoclonal antibodies to the monocytic markers CD172a, CD14, MHCII and CD163 to analyze the existence of monocyte subsets in camel blood.

The signal-regulatory protein alpha (SIRPalpha), also known as CD172a, which was highly expressed on camel monocytes, was used to identify total camel monocytes. This is in line with studies in the pig and bovines, where CD172a is characterized as a pan monocyte marker [6–8, 14, 21]. Although CD172a is also expressed at low levels on plasmacytoid DC [22, 23], CD172a-positive camel monocyte subsets are distinct from blood dendritic cells, which are negative for both CD14 and CD163 [24].

Staining with CD172a, CD14 and MHCII antibodies identified three CD172a-positve camel subsets: CD14^high^MHCII^low^ (subset mo-I), CD14^high^MHCII^high^ (subset mo-II) and CD14^low^MHCII^high^ monocytes (subset mo-III). A direct comparison of camel monocyte subsets with those in the human or bovine system is not possible due to the lack of camel-specific CD16 antibodies. Indirectly, based on their high CD14 and CD163 expression, their low MHCII expression together with their percentage in blood (87% of total monocytes), camel monocytes mo-I are very likely equivalent to bovine and human classical monocytes [7, 12]. Camel monocytes mo-II, due to their highest expression of MHCII and their high level expression of CD14 and CD163 can be suggested as equivalents to human and bovine intermediate monocytes. This is also supported by the anti-bacterial and inflammatory nature of mo-II cells, as they showed the highest phagocytosis activity among camel monocytes and ROS generation activity in comparison to the other two camel subsets. These functional activities are also comparable with the inflammatory nature of bovine intermediate monocytes [6, 11]. The low expression density of CD14 and CD163 and the high expression of MHCII on the camel mo-III monocytes strongly suggest that this camel monocyte subset represents a counterpart of bovine non-classical monocytes. This is supported by their highest expression of adhesion molecule leukocyte functional antigen 1 (LFA-1 or CD11a) and the low level expression density of the adhesion molecules CD18 and Mac1 (CD11b) on camel mo-III cells, which is comparable to the bovine system [7, 25]. In addition, the low phagocytic and ROS generation activity of camel mo–III monocytes is in line with findings reported for bovine non-classical monocytes [6].

The demonstrated phenotypical characteristics of camel monocytes seem to change during ontogeny since significant differences between monocytes of newborn and adult camels were obtained. Newborn camels showed significantly higher total monocyte numbers and numbers of mo-I and mo-III cells in blood whereas mo-II cells were less abundant in blood of newborns compared to adults.. Whether these differences are associated with a different equipment with chemokine receptors and hence a different migration behavior into tissues or whether this reflects differences in maturation/release processes from the bone marrow can only be hypothesized. The different expression densities of monocyte-related surface molecules between newborn and adult camels (Fig. 7) may support this idea. Altogether these findings demonstrate age related effects on the distribution and phenotype of camel monocyte subsets, which may contribute to an altered function of the newborn innate immune system [26].

## Conclusions

In summary, we identified three monocyte subsets in dromedary camel blood based on the surface expression of CD14, CD163 and MHCII: camel monocyte subset mo-I (CD14^high^MHCII^low^), camel monocyte subset mo-II (CD14^high^MHCII^high^) and camel monocyte subset mo-III (CD14^low^MHCII^high^). The analysis of phenotypic and functional properties suggests that camel monocyte subsets mo-I, mo-II and mo-III are counterparts of bovine classical, intermediate and non-classical monocytes respectively. Age related changes in camel monocyte numbers and phenotype were identified.

## Methods

### Animals and sample collection

Blood samples were collected from clinically healthy camels (*Camelus dromedaries)* at the Camel Research Center, King Faisal University, Saudi Arabia. The involved animals included sixty-two adult camels aged between four and ten years and twenty-three newborn calves (aged < four weeks). Blood was obtained by venepuncture of the *vena jugularis externa* into vacutainer tubes containing EDTA (Becton Dickinson, Heidelberg, Germany). All experimental procedures and management conditions used in this study were approved by the Ethics Committee at King Faisal University, Saudi Arabia (Permission number DSR 1811001).

### Hypotonic lysis and separation of whole blood leukocytes

Separation of whole camel leukocytes was done after hypotonic lysis of blood erythrocytes [20]. Briefly, blood was suspended in distilled water for 20 s and double concentrated PBS was added to restore tonicity. This was repeated until complete erythrolysis. Separated cells were finally suspended in MIF buffer (PBS containing bovine serum albumin (5 g/L) and NaN_3_ (0.1 g/L)) at 5 × 10^6^ cells/ml. The mean viability of separated cells was evaluated flow cytometrically by dye exclusion (propidium iodide; 2 μg/ml, Calbiochem, Germany) and consistently > 95%.

### Monoclonal antibodies

Monoclonal antibodies used in this study are listed in Table 2.

**Table 2:** List of antibodies

Antigen	Antibody clone	Labelling	Source	Isotype
CD172a	DH59b	–	WSU	mIgG1
CD14	TÜK4	PerCP	Biorad	mIgG2a
CD14	TÜK4	APC	Biorad	mIgG2a
CD163	LND68A	–	WSU	mIgG1
MHCII	TH81A5	–	WSU	mIgG2a
CD11a	G43-25B	PE	BD	mIgG2a
CD11b	ICRF44	PE-Cy7	BD	mIgG1
CD18	6.7	FITC	BD	mIgG1
mIgG2a	polyclonal	PE	Invitrogen	gIgG
mIgG1	polyclonal	FITC	Invitrogen	gIgG

*Ig* Immunoglobulin; *m* mouse; *MHC-II* Major Histocompatibility Complex class II, *g* goat, *WSU* Washington State University

### Cell labeling and flow cytometric analysis of camel blood monocytes

The identification of camel monocyte subsets and the analysis of the subset-specific expression pattern of monocytic markers and adhesion molecules were performed after direct and indirect labeling of cells with surface molecule-specific antibodies and flow cytometrical analysis [27]. Separated camel leukocytes (5 × 10^6^ cells / ml) were incubated in 96 well round-bottom microtiter plates (1 × 10^6^ / well; 20 min; 4 °C), in a three-step staining process, with monoclonal antibodies specific for CD172a, MHCII and CD163 (in the following two combinations: CD172a; MHCII; CD14 and CD163; MHCII; CD14) or with isotype control antibodies in PBS containing bovine serum albumin (5 g / L) and NaN_3_ (0.1 g / L). After incubation, cells were washed twice and incubated with mouse secondary antibodies IgG1, IgG2a (BD) labelled with different fluorochromes. In a third labelling step, directly labeled monoclonal antibodies to CD14, CD11a, CD11b, and CD18 were added. Finally, cells were washed and analyzed by flow cytometry. For each measurement 100,000 events were acquired. Flow cytometric data were analyzed with the software *FlowJo* version 10 (FLOWJO LLC). After the microscopic estimation of the total leukocyte count (using Türk Solution and Neubauer counting chamber), absolute cell count (cell per ml blood) of monocyte subsets was evaluated.

### Analysis of reactive oxygen species (ROS) generation

ROS generation was performed in 96-well round-bottom microtiter plates (Corning, NY, USA) as described earlier [28] with modifications. Separated camel leukocytes (1 × 10^6^ / well) in RPMI medium were incubated with heat killed *Staphylococcus aureus* (50 bacteria/cell) for 20 min (37 °C, 5% CO_2_). For the detection of ROS, dihydrorhodamine (DHR) 123 (Mobitec, Goettingen, Germany) was added to the cells (150 ng / ml final). To identify monocyte subsets, cells were labeled with monoclonal antibodies to CD14 and MHCII (see above). After washing, cells were analyzed by flow cytometry (FACSCalibur, Becton Dickinson Biosciences, San Jose, California, USA). The relative amount of generated ROS was determined by the mean green fluorescence intensity of gated monocyte subsets (based on CD14 and MHCII expression) after acquisition of 100,000 events (*n* = 15 animals).

### Phagocytosis assay

Heat killed *Staphylococcus aureus* (*S. aureus*) bacteria (Pansorbin, Calbiochem, Merck, Nottingham, UK) were labeled with fluoresceinisothiocyanate (FITC, Sigma-Aldrich, St. Louis, Missouri, USA). FITC-conjugated and heat killed *S. aureus* bacteria were suspended in RPMI medium and adjusted to 2 × 10^8^ bacteria / ml. Separated camel leukocytes were plated in 96 well plates (1 × 10^6^/well) and incubated (37 °C, 5% CO_2_) with labeled bacteria (50 bacteria / cell) for 40 min (37 °C, 5% CO_2_). To identify monocyte subsets, cells were labeled with monoclonal antibodies to CD14 and MHCII (see above). After washing, cells were analyzed by flow cytometry (FACSCalibur, Becton Dickinson Biosciences, San Jose, California, USA). After washing, cells were analyzed by flow cytometry (FACSCalibur, Becton Dickinson Biosciences, San Jose, California, USA). For each monocyte subset (based on their CD14 and MHCII expression), phagocytosis-positive cells were defined as the percentage of green fluorescing cells among total cells (*n* = 15 animals). Phagocytosis capacity (as an indicator for the number of bacteria ingested by each monocyte) was defined as the mean green fluorescence intensity of gated phagocytosis-positive monocyte subset.

### Statistical analyses

For statistical analysis, the software Prism (GraphPad) was used. Results are expressed as means ± S.E. of the mean (SEM). For the comparison between means of two groups, the t-test was used. Differences between means of more than two groups were tested with one-factorial analysis of variance (ANOVA) and Bonferroni’s correction for normally distributed data. Results were considered statistically significant at a *p*-value of less than 0.05.

## Data Availability

The datasets used and/or analysed during the current study are available from the corresponding author on reasonable request.

## Abbreviations

CD: Cluster of differentiation
FITC: Fluorescein isothiocyanate
LFA-1: Leukocyte functional antigen 1
MFI: Mean fluorescence intensity
MHC: Major histocompatibility complex
mo: Monocyte
ROS: Reactive oxygen species
SIRPalpha: Signal-regulatory protein alpha
